# Case report: A case of Rabson–Mendenhall syndrome: long-term follow-up and therapeutic management with empagliflozin

**DOI:** 10.3389/fgene.2024.1414451

**Published:** 2024-06-24

**Authors:** R. Foglino, F. Barbetti, E. Morotti, V. Castorani, A. Rigamonti, G. Frontino, G. Barera, R. Bonfanti

**Affiliations:** ^1^ Pediatric Diabetes Unit IRCCS Ospedale San Raffaele, Vita Salute San Raffaele University, Milan, Italy; ^2^ Monogenic Diabetes Clinic, Unit of Endocrinology and Diabetes, Bambino Gesù Children Hospital IRCCS, Rome, Italy

**Keywords:** Rabson–Mendenhall syndrome, severe insulin resistance, sodium–glucose cotransporter 2 inhibitors, glycemic control, insulin receptor mutations, metabolic syndrome, insulin signaling pathways

## Abstract

**Background:** Rabson–Mendenhall syndrome (RMS), a rare disorder characterized by severe insulin resistance due to biallelic loss-of-function variants of the insulin receptor gene (INSR), presents therapeutic challenges (OMIM: 262190). This case study explores the efficacy of adjunctive therapy with sodium–glucose cotransporter 2 inhibitors (SGLT2is) in the management of RMS in an 11-year-old male patient with compound heterozygous pathogenic variants of INSR.

**Methods:** Despite initial efforts to regulate glycemia with insulin therapy followed by metformin treatment, achieving stable glycemic control presented a critical challenge, characterized by persistent hyperinsulinism and variable fluctuations in glucose levels. Upon the addition of empagliflozin to metformin, *notable* improvements in glycated hemoglobin (HbA1c) and time in range (TIR) were observed over a 10-month period.

**Results:** After 10 months of treatment, empagliflozin therapy led to a clinically meaningful reduction in HbA1c levels, decreasing from 8.5% to 7.1%, along with an improvement in TIR from 47% to 74%. Furthermore, regular monitoring effectively averted normoglycemic ketoacidosis, a rare complication associated with SGLT2 inhibitor therapy.

**Conclusion:** This case highlights the potential of SGLT2i as adjunctive therapy in RMS management, particularly in stabilizing glycemic variability. However, further research is warranted to elucidate the long-term efficacy and safety of this therapeutic approach in RMS and similar insulin resistance syndromes.

## Introduction

Rabson–Mendenhall syndrome (RMS) represents a multisystemic disorder manifesting through a wide range of signs and symptoms. First described by Rabson and Mendenhall in 1956, RMS is a rare form of congenital severe insulin resistance (c.SIR) caused by biallelic, loss-of-function variants of the insulin receptor (*INSR*; 19p13.3-p13.2) (Taylor et al., 1992). The exact prevalence of RMS has not been established, but recent data on c. SIR linked to *INSR* mutations, that include RMS and Donohue syndrome (DS, also known as leprechaunism), set the incidence to ≈1,200,000–1,300,000 live births (Rapini et al., 2024). Prognosis *quoad vitam* of *INSR*-c. SIR depends on the extent of insulin action impairment (Longo et al., 2002), with patients clinically classified as DS being prone to fatal outcomes before the age of 1 year (Longo et al., 2002; Grasso et al., 2013; Rapini et al., 2024). Both clinical subtypes are characterized by intrauterine growth restriction (IUGR) with RMS patients showing often times short stature later in childhood. Coarse facies, abdominal distension, adipose tissue hypotrophy along with cutaneous manifestations such as acanthosis nigricans, and hypertrichosis are associated with both DS and RMS (Taylor et al., 1992; Grasso et al., 2013), while early dentition, gingival hyperplasia, and nephrocalcinosis have been mainly described in RMS (Taylor et al., 1992; Cochran et al., 2005; Grasso et al., 2013). Very high insulin levels with alternation of hypoglycemia and hyperglycemia are found in DS and RMS during the neonatal period and beyond. However, in patients with RMS, hypoglycemic episodes usually subside overtime with a transition to constant hyperglycemia and high glycated hemoglobin. The treatment of insulin resistance syndromes represents a conundrum and a challenge with most patients experiencing poor glycemic control and early diabetic complications. Early attempts to treat patients with *INSR*-c. SIR with an extremely high insulin dose either alone (Cochran et al., 2005) or in combination with metformin (Abdelaziz et al., 2016) or metformin plus rosiglitazone (Vestergaard et al., 2001; Musso et al., 2004) were disappointing. However, in an RMS patient, multidrug therapy to a final dose of 45 mg/d of pioglitazone, 2 g/d of metformin, 100 mg/d of vildagliptin, and 150 mg/d of acarbose caused an HbA1c reduction from 12% to 7% over a period of 16 months (Moreira et al., 2010). Subcutaneous injection of human recombinant IGF1 (rhIGF1, mecasermin) and human methionyl-leptin (metreleptin) have been employed with better outcomes on glycemic control in subjects with *INSR*-c. SIR (Okawa et al., 2022; Semple et al., 2023). However, doubts have been cast about a possible interference of metreleptin with growth hormone therapy in these subjects (Okawa et al., 2022). A new therapeutic approach for Rabson–Mendenhall syndrome involves the off-label use of sodium–glucose cotransporter 2 inhibitor (SGLT2i) tablets, originally developed for type 2 diabetes treatment. SGLT2 is responsible for the majority of glucose reabsorption in the kidneys, accounting for about 90% of the glucose reabsorption from the glomerular filtrate. It works by coupling the movement of sodium ions (Na⁺) and glucose molecules across the luminal membrane of the renal tubular cells. Specifically, for every one molecule of glucose reabsorbed, two sodium ions are cotransported into the cell against their concentration gradient. SGLT2 inhibitors, such as empagliflozin, canagliflozin, and dapagliflozin, selectively block the activity of SGLT2. By doing so, they prevent the reabsorption of glucose from the renal tubules back into the bloodstream. This leads to increased urinary glucose excretion (glucosuria) and consequently lowers blood glucose levels. SGLT2i treatment of *INSR*-c. SIR is a rational approach because it circumvents the problem posed by the severely impaired insulin action in skeletal muscle and liver and adipose tissues that jeopardizes treatments based on insulin, insulin secretagogues, and incretin mimetics in these patients. When initiating SGLT2i, it is crucial to consider the risks associated with drug intake despite its potential benefits. Normoglycemic ketoacidosis, a serious side effect, can occur despite normal blood sugar levels. This condition, marked by elevated blood ketone levels, demands prompt treatment to avert complications. Moreover, in conditions like RMS, where weight gain is crucial, the weight loss associated with SGLT2 inhibitors might be unfavorable. Hence, vigilant monitoring of capillary ketone levels and a periodic assessment of growth parameters are imperative to ensure safe management of SGLT2 inhibitor therapy in RMS patients. Additionally, further side effects such as urinary tract infections due to persistent glucosuria should be considered, necessitating regular urine tests. Volume depletion is another concern; therefore, in cases of gastroenteritis, given the known susceptibility to dehydration in pediatric patients, therapy should be suspended. In the present investigation, we evaluated the effectiveness of SGLT2i in reducing hemoglobin A1C and improving the time in range (TIR) in an 11-year-old male patient with RMS associated with compound heterozygous *INSR* pathogenic variants (Grasso et al., 2013).

## Clinical case

**FIGURE 1:**
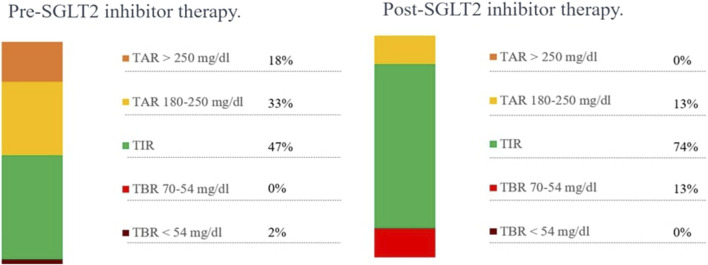
Metabolic monitoring before and after 10 months of SGLT2 inhibitor therapy. TAR, time above range; TIR, time in range; TBR, time below range.

**FIGURE 2:**
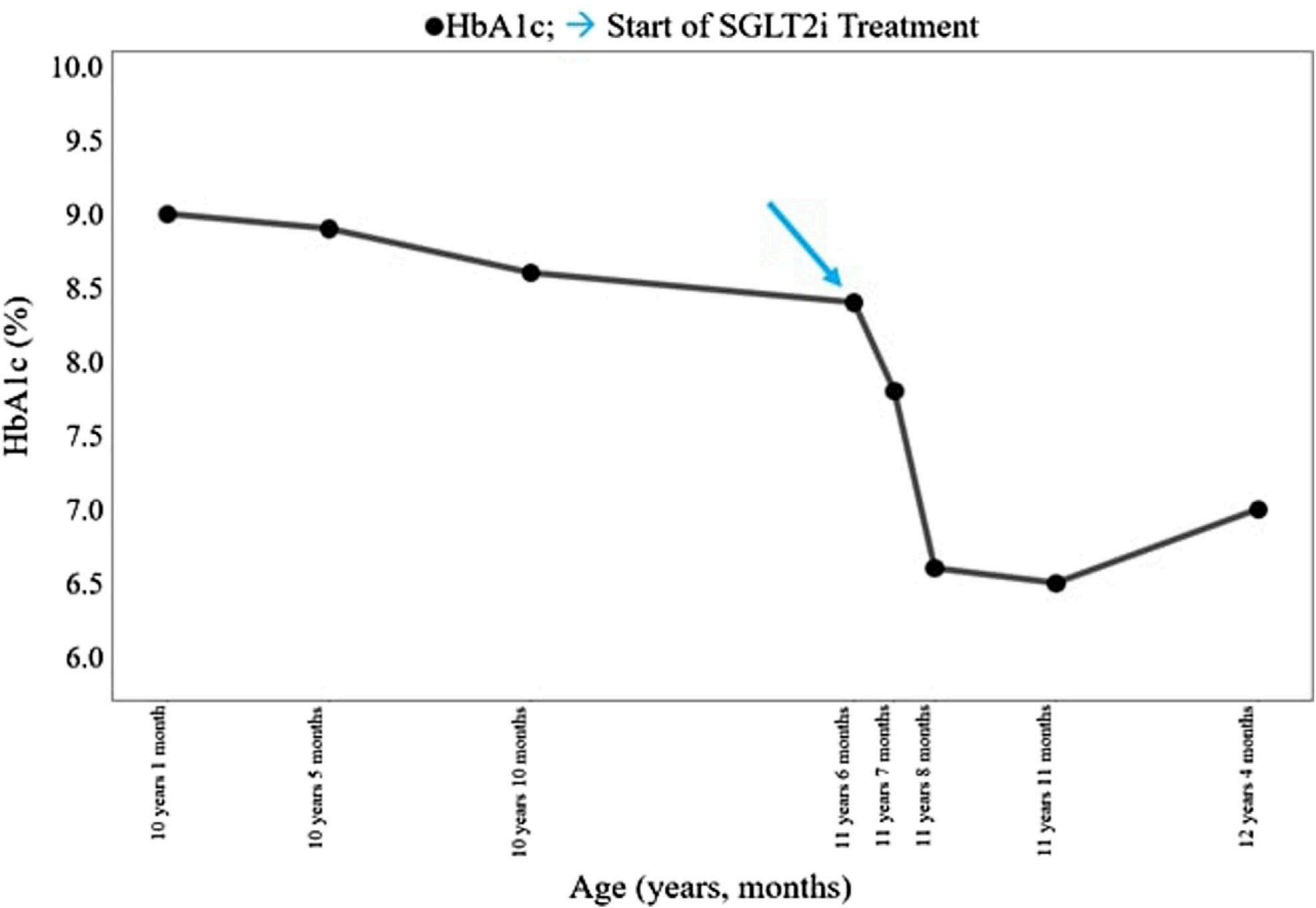
Clinical course of the patient before and after SGLT2i treatment. HbA1c, hemoglobin glycated (%). SGLT2i, sodium–glucose cotransporter 2 inhibitor. → start of SGLT2i treatment.

**FIGURE 3:**
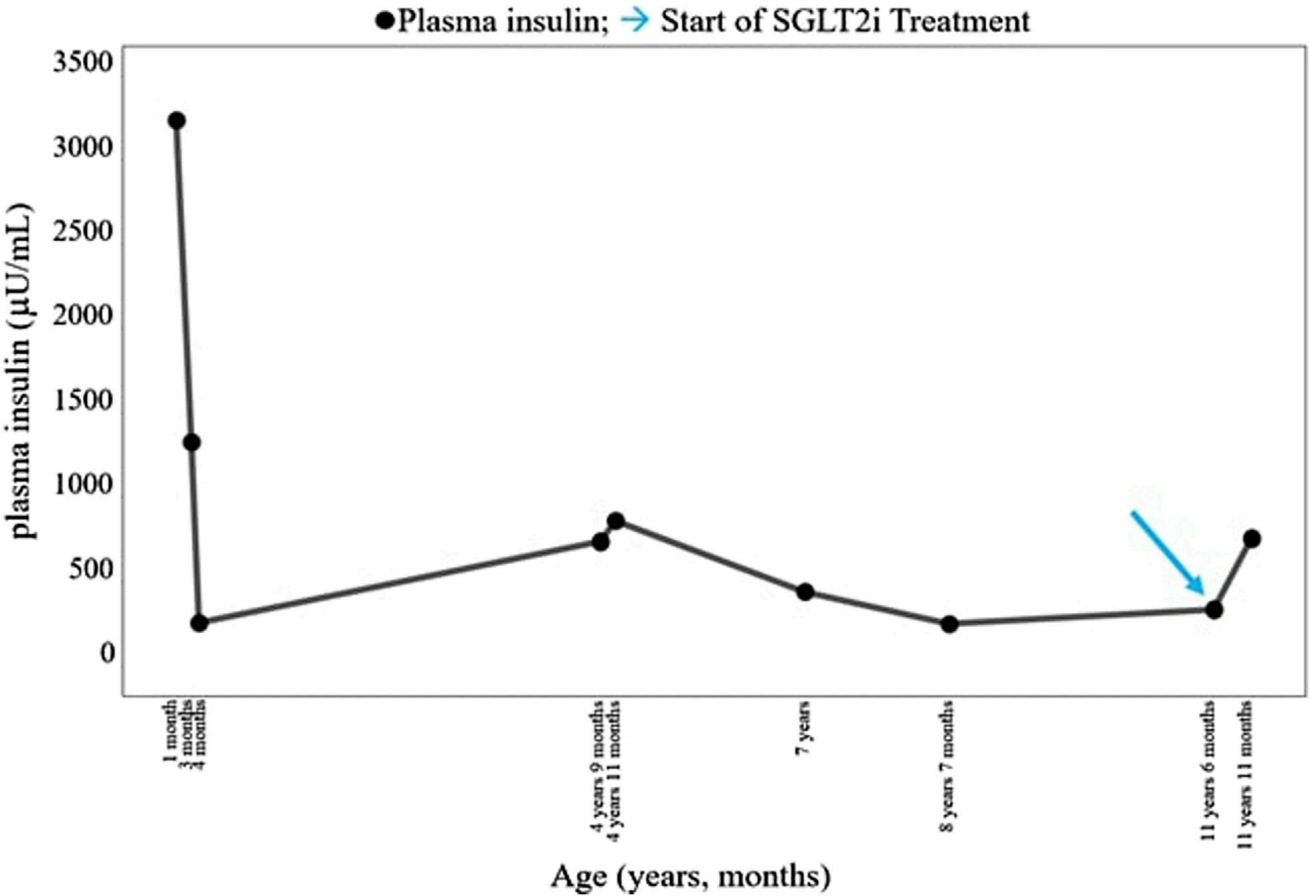
Course of the patient’s plasma insulin (µU/mL). SGLT2i, sodium–glucose cotransporter 2 inhibitor. → start of SGLT2i treatment.

The patient was born at term to non-consanguineous parents with a birth weight of 2,225 g (third percentile), a length of 45.5 cm, and a head circumference of 31.5 cm, and classified small for gestational age (SGA). Family history revealed renal glycosuria in the paternal branch of the family. On day 16, the patient was brought to the emergency room because of lack of appetite, hyporeactivity, and the presence of oral candidiasis. The proband presented with a blood glucose level of 621 mg/dL without diabetic ketoacidosis [values from the blood gas analysis pH: 7.5, HCO_3_ 22.2 mmol/L, BEB (base excess in blood) −2.4 mmol/L, blood glucose: 268 mg/dL]; he was negative to type 1 diabetes-associated autoantibodies (ZnT8A, anti-pancreatic islet antibodies-ICA, GADA, IA-2A, and IAA). Plasma insulin and c-peptide values were 3,234.4 µU/mL and 42.3 ng/mL, respectively. Genetic analysis (Sanger method) revealed compound heterozygous pathogenic variants of the insulin receptor gene (INSR) c.121G>A, p. Arg41Trp, and c.1268 + 2T>C) (Grasso et al., 2013) consistent with the diagnosis of c. SIR. Initial diabetes management was based on insulin therapy. The dose was swiftly scaled up to 35 units (approximately 10 U/Kg/day), with glucose levels fluctuating between 150 and 250 mg/dL before feeding and 300–400 mg/dL after feeding. Because of the poor glycemic control, insulin therapy was stopped at 1 month of age and metformin was started, administered as galenic preparation via oral suspension. This treatment initially improved the glycemic profile; however, metformin was discontinued at 5 months of age in consideration of low blood glucose values, along with the persistence of marked hyperinsulinism (257 µU/mL). When 3 years old, while free of therapy, data download revealed marked glycemic variability, with an average glucose level of 148 mg/dL ± 67 mg/dL, a minimum value of 42 mg/dL, and a maximum of 319 mg/dL. At this time, the patient was clinically classified with RMS. Therapy with metreleptin was contemplated at the age of 7 years (HbA1c 58 mmol/mol, 7.5%) but was not initiated due to normal leptin levels for BMI, along with practical challenges accessing, affording, and administering the medication, and given the expected partial benefit. At 8 years and 8 months (HbA1c 71 mmol/mol, 8.6%), metformin was reintroduced at 500 mg daily and then was gradually increased to 500 mg t. i.d. in January 2022. At the age of 11 years and 6 months, owing to the persisting hyperinsulinism (335 µU/mL) and high glycated hemoglobin levels (69 mmol/mol or 8.5%), off-label therapy with SGLT2 inhibitors was proposed to the patient’s parents. Empagliflozin was added to metformin (500 mg t. i.d.) starting at the dose of 2.5 mg/d that was increased to 5 mg/day (Galderisi et al., 2022) in 20 days. Regular ketone tests and urine examinations were prescribed to monitor albuminuria, creatininuria, calciuria, phosphaturia, and the emergence of possible infections. A glucose sensor (Libre 3) was used for continuous glucose monitoring. Capillary ketonemia was monitored at home daily for the initial 15 days and subsequently once a week for the following 3 months. The mean value of ketonemia was 0.3 mmol/L, with a maximum value of 0.4 mmol/L, consistently within physiological levels (<0.6 mmol/L). The urine tests have never revealed any issues. There were no reported values exceeding 0.6 mmol/L. At the onset of therapy, the height, initially recorded at 142.4 cm (−0.72 SDS), increased to 147 cm (−0.75 SDS) after 10 months. Pre-therapy weight was 29.85 kg (−1.72 SDS), while after 10 months, it measured 31 kg (−2.01 SDS). Considering the effects of SGLT2i, particularly on weight, and the underlying condition of RMS, auxological parameters remained stable, albeit consistently at lower percentiles. Thereafter, 10 months after the initiation of therapy, the time in range (TIR) increased from 47% (with metformin monotherapy) to 63% with empagliflozin 2.5 mg (first month of therapy), reaching 74% with empagliflozin 5 mg. (Figure 1). Contrary, the time below range (TBR) increased from 0% to 13%. Nevertheless, no hypoglycemic episodes were reported. Glycated hemoglobin decreased from 8.5% to 7.1% during the same period, indicating a *noteworthy* improvement in glycemic control (Figure 2). However, plasma insulin levels remained elevated (Figure 3).

## Discussion/conclusion

This clinical case offers an in-depth insight into RMS, highlighting the therapeutic challenges associated with the glycemic variability observed in the patient. Over time, due to the impaired activity of the insulin receptor, stable hyperglycemia ensued. In this phase of disease, the introduction of SGLT2i had a positive impact on glycated hemoglobin and the quality of life, suggesting potential benefits in the long-term management of RMS. The increase in TBR aligns with the clinical characteristics of RMS, wherein patients’ glycemic profiles exhibit considerable variability and fasting hypoglycemia. Therefore, as the average blood glucose decreases, it is predictable to observe a rise in the percentage of hypoglycemic values. Additionally, another advantage of using SGLT2i is their potential to reduce the risk of nephrocalcinosis (Paik et al., 2024), a likely manifestation in affected patients. Of importance, regular monitoring of ketonemia and urine examinations is essential for the prevention of normoglycemic ketoacidosis, a rare, but severe complication of SGLT2i therapy (Bonora et al., 2018). Our report confirms that the use of SGLT2i in patients with RMS (Galderisi et al., 2022) is a suitable option as an add-on to metformin that—when used as monotherapy—failed to obtain any improvement in the metabolic control of this individual. Similar results have been reported in an RMS patient whose glycemic control deteriorated at the age of 11 years. In this case, empagliflozin 5 mg was added to insulin and metformin, resulting in a HbA1c decrease from 10.5% to 7.7% and simultaneous reduction of 30% of the insulin dose (dos et al., 2021). In a case with monoallelic *INSR*-SIR type, the addition of ipragliflozin (50 mg) to metformin (2,250 mg/d) and acarbose (300 mg/d) leads to the decrease of HbA1c from 10% to 7.2% (Nagashima et al., 2020). Of note, HbA1c was maintained around 8% thereafter, with no adverse effects (Table 1). In conclusion, SGLT2is seem to be a valuable option for the treatment of INSR-c. SIR and possibly type-A SIR, when patients show stable diabetes. In addition, taking into account that SGLT2is do not cause hypoglycemia (Audet et al., 2021), the use of drugs of this class to prevent beta-cell failure often observed in *INSR*-c. SIR should be evaluated. However, long-term efficacy of SGLT2is in the aforementioned conditions remains to be established.

## Data Availability

The original contributions presented in the study are included in the article/Supplementary Material; further inquiries can be directed to the corresponding authors.

## Supplementary material

SUPPLEMENTARY TABLE S1 SGLT2i therapy in cases with Rabson–Mendenhall syndrome.
