# Supplementary material for: Case report: A case of Rabson–Mendenhall syndrome: long-term follow-up and therapeutic management with empagliflozin
Source: Front Genet. 2024 Jun 24;15:1414451. doi: 10.3389/fgene.2024.1414451 (PMC11228259; doi:10.3389/fgene.2024.1414451)
Supplement: Supplementary file 2 [file DataSheet1.docx]

**CARE CHECKLIST**

**1. Title –** A Case of Rabson-Mendenhall Syndrome: Long-Term follow-up and Therapeutic Management with Empagliflozin - “Case Report”.

**2. Key Words -** Rabson Mendenhall Syndrome, severe insulin resistance, sodium-glucose co-transporter 2 inhibitors, insulin receptor mutations, case report.

**3. Abstract**

Background: Rabson Mendenhall Syndrome (RMS), a rare disorder characterized by severe insulin resistance due to biallelic loss-of-function variants of the insulin receptor gene (INSR), presents significant therapeutic challenges (OMIM: 262190). This case study explores the efficacy of adjunctive therapy with sodium-glucose co-transporter 2 inhibitors (SGLT2i) in the management of RMS in an 11-year-old male patient with compound heterozygous pathogenic variants of INSR.

Methods: Despite initial efforts to regulate glycemia with insulin therapy followed by metformin treatment, achieving stable glycemic control presented a significant challenge, characterized by persistent hyperinsulinism and variable fluctuations in glucose levels. Upon the addition of empagliflozin to metformin, significant improvements in glycated hemoglobin (HbA1c) and Time in Range (TIR) were observed over a ten-month period.

Results: Empagliflozin therapy resulted in a significant reduction in HbA1c levels and an improvement in Time in Range (TIR). Moreover, regular monitoring successfully prevented normoglycemic ketoacidosis, a rare complication associated with SGLT2i therapy.

Conclusion: This case highlights the potential of SGLT2i as adjunctive therapy in RMS management, particularly in stabilizing glycemic variability. However, further research is warranted to elucidate the long-term efficacy and safety of this therapeutic approach in RMS and similar insulin resistance syndromes.

**4. Introduction -** This case presents the innovative use of sodium-glucose co-transporter 2 inhibitors (SGLT2i) in treating Rabson Mendenhall Syndrome (RMS), a rare form of severe congenital insulin resistance (c.SIR). RMS is caused by biallelic variants of the insulin receptor gene (INSR) and is associated with significant challenges in glycemic control. Traditionally, treatment for RMS has involved high doses of insulin combined with other medications, but with limited success in achieving stable glycemic control. This case introduces the use of SGLT2i, originally developed for type 2 diabetes, which act by inhibiting glucose reabsorption in the kidneys. The introduction of this therapy represents a promising option to improve glycemic control in patients with RMS.

**5. Patient Information -** The patient was an 11-year-old male born at term to non-consanguineous parents with a birth weight of 2225 g (3rd percentile), a length of 45.5 cm, and a head circumference of 31.5 cm, classified as small for gestational age (SGA). On the sixteenth day of life, the patient was brought to the emergency room because of lack of appetite, hyporeactivity, and the presence of oral candidiasis. The patient's general blood tests were within normal limits, but plasma insulin and c-peptide values were significantly elevated. Genetic analysis revealed compound heterozygous pathogenic variants of the insulin receptor gene (INSR) consistent with the diagnosis of severe insulin resistance (SIR). Initial diabetes management was based on insulin therapy followed by metformin treatment, which resulted in significant improvements in the glycemic profile. However, metformin was discontinued at 5 months of age due to low blood glucose values and persistent hyperinsulinism. At 8 years and 8 months, metformin was reintroduced and adjusted over time.

**6.Clinical Findings -** The patient presented with symptoms of lack of appetite, hyporeactivity, and oral candidiasis on the sixteenth day of life. Laboratory tests revealed significantly elevated plasma insulin and c-peptide levels. Genetic analysis confirmed compound heterozygous pathogenic variants of the insulin receptor gene consistent with severe insulin resistance. Of note, patient's multisystemic manifestations over the years included cardiac involvement with ST-segment elevation in V1-V2 derivations, resembling that seen in Brugada syndrome type 1. In addition, numerous perforations of the tympanic membrane occurred following infectious processes (middle ear infections), a typical finding of *INSR*-c.SIR. Throughout the patient's medical history, there were notable fluctuations in glycemic control despite various therapeutic interventions.

**7. Timeline**

| Day 16 | Patient presents with lack of appetite, hyporeactivity, and oral candidiasis; laboratory tests reveal significantly elevated plasma insulin and c-peptide levels. |
| --- | --- |
| 1 month | Insulin therapy stopped and metformin started. |
| 5 months | Metformin stopped in consideration of low blood glucose values. |
| 3 years old | Glucose sensor (Freestyle Libre) applied to the patient, revealing significant glycemic variability. |
| 8 years and 8 months | Metformin reintroduced 500 mg q.d. |
| 9 years and 10 months | Metformin dose was adjusted to 500 mg b.i.d |
| 10 years and 11 months | Metformin dose was adjusted to 500 mg t.i.d |
| 11 years and 7 months | SGLT2 inhibitor therapy initiated. |

**8. Diagnostic Assessment -** Diagnostic methods included laboratory testing, genetic analysis, and continuous glucose monitoring. Challenges in diagnosis and management included persistent hyperinsulinism and glycemic variability. The primary diagnosis was severe insulin resistance due to compound heterozygous pathogenic variants of the insulin receptor gene.

**9. Therapeutic Intervention -** Therapeutic interventions included insulin therapy, metformin treatment, and the addition of empagliflozin as adjunctive therapy. Empagliflozin was initiated at a low dose and gradually titrated up over a ten-month period.

**10. Follow-up and Outcomes -** Clinician- and patient-assessed outcomes included glycated hemoglobin levels, Time in Range (TIR), and plasma insulin levels. Empagliflozin therapy resulted in significant reductions in HbA1c levels and improvements in TIR. Regular monitoring successfully prevented normoglycemic ketoacidosis. Adverse events were not reported.

**11. Discussion -** This case report illustrates the therapeutic challenges associated with Rabson Mendenhall Syndrome (RMS) and the potential role of sodium-glucose co-transporter 2 inhibitors (SGLT2i) as adjunctive therapy in managing severe insulin resistance. The addition of empagliflozin to metformin resulted in significant improvements in glycemic control, highlighting the potential of SGLT2i in stabilizing glycemic variability. However, further research is warranted to establish the long-term efficacy and safety of this therapeutic approach in RMS and similar insulin resistance syndromes.
This case underscores the therapeutic complexity of Rabson Mendenhall Syndrome (RMS) and the significant challenges in achieving stable glycemic control in affected individuals. Despite initial treatment with insulin and metformin, persistent hyperinsulinism and glycemic variability persisted, necessitating alternative therapeutic strategies. The addition of empagliflozin, an SGLT2 inhibitor, to the patient's regimen resulted in notable improvements in glycated hemoglobin levels and Time in Range (TIR), highlighting its potential as a promising adjunctive therapy in RMS management. Regular monitoring played a crucial role in mitigating potential complications associated with SGLT2 inhibitor therapy. This case underscores the importance of individualized treatment approaches and ongoing research to optimize outcomes in patients with RMS and similar insulin resistance syndromes.

**12. Patient Perspective –** The patient's perspective on the treatment received was not provided in this case report.

**13. Informed Consent –** Informed consent was obtained from the patient's parents for the publication of this case report.
